# The Articulation of the Indigenous Peoples of Brazil in Facing the Covid-19 Pandemic

**DOI:** 10.3389/fsoc.2021.611336

**Published:** 2021-03-17

**Authors:** Luciana Leite da Silva, Patrícia Emanuelle Nascimento, Ordália Cristina Gonçalves Araújo, Tamiris Maia Gonçalves Pereira

**Affiliations:** Department of History, Federal University of Goiás, Goiânia, Brasil

**Keywords:** COVID-19, indigenous, Brazil, body, territory

## Abstract

This article aims to analyze how the indigenous communities of Brazil have organized autonomous actions and strategies to confront the Covid-19 pandemic based on the articulation among their own historical experiences, their health conceptions, partnerships with scientific communities and other segments of society that support the indigenous struggle. The research articulates the political and theoretical modernity/coloniality/decoloniality movement with indigenous experiences and conceptions of health, body/spirituality and territory. For this task, we adopted an undisciplined methodology based on conversation, solidarity and analysis of discussions, sites, lives, bibliographic productions and official documents prepared by indigenous organizations and partner entities. The research has pointed out that the situation of greater vulnerability of indigenous populations is not only due to biological factors. Also, indigenous people have denounced the invasion of their territories, racism, the lack of sanitation policies, food insecurity, the circulation of people not belonging to the community (missionaries, miners, loggers, army), the difficult access to hospitals and the precariousness of the necessary resources for individual and collective asepsis have worsen the spread and lethality of the virus. Likewise the current indigenous struggle in this pandemic scenario, this article is not limited to a health discussion, yet it aims to contribute to think about the relationship between the pandemic and the dissemination of anti-democratic policies that simultaneously affect the right to health and the territory of these populations.

## Introduction

April 1, 2020 marks the official date of the first Covid-19 registration case among Brazilian indigenous peoples. On June 20, COIAB (Coordination of Indigenous Organizations of the Brazilian Amazon) and IPAM (Institute of Environmental Research of the Amazon) released a study indicating that the mortality rate by coronavirus among indigenous people is 150% higher than the Brazilian average, that is to say, among indigenous people the rate is 6.8%, while the average for Brazil is 5% (Fellows et al., 2020). On September 7, the APIB (Articulation of Indigenous Peoples of Brazil) updates the data, indicating the number of 30,218 indigenous people infected, 786 deaths; 156 people affected by the pandemic (APIB 2020a).

These facts have exposed a range of problems that will be here addressed. Is the greatest vulnerability of indigenous populations due only to biological factors? What are the great challenges and how have these populations been organized to face them in the current context?

Our hypothesis is that the worsening of indigenous vulnerabilities results not only from the greater susceptibility to exogenous diseases, but also from social and economic conditions and the difficulty of access to the health system (COVID-19 pandemic and collaborators, 2005). All these conditions have historically arisen from the process of coloniality established since the 16th century, going through centuries of violation of indigenous rights by the State and its agents (colonizers, sertanists, missionaries, indigenists). The roots planted in this process are currently reflected in the continuity of the invasion of their territories, the precarious access to health services (hampered by logistic distance or by the reduced number of staff), and the institutional racism perpetrated in political actions of neglect of indigenous rights or encouragement to their disrespect.

As David Kopenawa states "all this destruction is not our mark, it is the footprint of the whites, your footprint on the earth" and "[We] Yanomami, the inhabitants of the forest, do not fill the earth with xawara smoke-epidemias" (Yanomani 2011, 21).

This trail full of ontological and epistemic racism is a producer of vulnerabilities and it has always been accompanied by various diseases such as measles, smallpox, rubella and flu. Notwithstanding, resistance has always been a response to the colonizing project of violation and destruction of indigenous peoples in Brazil. In the midst of a pandemic of high and rapid contagion such as that of Covid-19, the indigenous people find themselves facing a new historical crossroads. On one hand, the Covid-19 reveals even more the racism of the Brazilian State and its necropolitics with a Federal Government that proposes assumedly anti-indigenous agendas. On the other hand, the pandemic threatens the ancestral memory with the elderly as a group at risk. It is based on orality, with the elderly being the guardians of culture.

Based on this scenario, it is considered the relevance of monitoring the role that the State has been playing in the executive and legislative powers of government, as well as in public bodies such as the National Indigenous Foundation (FUNAI), the Special Indigenous Health Secretariat (SESAI) and the Special Indigenous Health Districts (DSEIs). Also, we will call the attention to how the indigenous social movements have lobbied the Federal Public Ministry and the Supreme Court of Justice to guarantee emergency access to indigenous health, both for those who live in their villages (homologated and non-homologated territories) and those who live in urban centers.

Once it a present time history, the analysis of the impacts of the Covid-19 pandemic among the indigenous peoples in Brazil demands the use of certain sources as: virtual meetings of the Mixed Parliamentary Front in Defense of the Rights of Indigenous Peoples; articles published by research institutes linked to the defense of native peoples; research coordinated by the Articulation of Indigenous Peoples of Brazil (APIB); protocolled documents in legislative bodies of the Federation; bibliography of indigenous authorship.

It is noteworthy that the methodology adopted by APIB will be an important guiding principle for this article. In a clear confrontation with the current indigenous policy adopted by the Brazilian State, APIB refuses to use the data collected under the guidance of the Ministry of Health, SESAI and the DSEIs that disregard in their statistical surveys the indigenous people who do not live in villages and in homologated lands. In this sense, the documentary corpus presented here is made up of diversified sources, whether bibliographic from research that investigates indigenous issues and, therefore, already consolidated in the academic environment along with virtual sources.

We propose to make use of the concept of interculturality in the analysis of these sources, treating them as "a space of negotiation and translation where social, economic and political inequalities, relations and power conflicts in society are not kept hidden, but recognized and confronted" (Walsh, 2001, 11).

## Materials and Methods

It is important to point out that the methodology adopted by APIB will be an important guideline for this article by reason of it is undisciplined, according to Haber and Nometodology (2011). It means to base the analyses on solidarity and conversation with indigenous peoples, generally disregarded by the Western methodology. In a clear confrontation with the current indigenist policy adopted by the Brazilian State, the APIB refuses to use the data collected under the guidance of the indigenous people who do not reside in villages and on approved lands. In this sense, the data presented here are composed of the "Indigenous grassroots organizations of the APIB, Covid-19 Confrontation Fronts that collaborate with the APIB, SESAI, Municipal and State Health Secretariats and the Federal Public Ministry" (APIB 2020a).

In this case, the sources produced as lives are highlighted, such as the meetings of the Committee of the Mixed Parliamentary Front in the Law of Indigenous Peoples, the radio sources (Radio Program COIAB), the documents written by the indigenous people (Indigenous Women March Final Document Letter by Indigenous Peoples from Around the World) or official documents (reports from the Public Ministry and the Ministry of Health) and journalistic sources (El País and Nexo). Thus, the data presented here are composed by the “APIB-based indigenous organizations, Fronts facing the Covid-19 that collaborate with APIB, SESAI, Municipal and State Health Secretariats and the Federal Public Ministry” (APIB 2020a) and also by official, historical, anthropological and journalistic data that emphasize the situation of indigenous peoples in facing pandemic contexts such as that of 2020. Therefore, data regarding the incidence of Covid-19 are collected in materials produced by indigenous organizations and published in digital format on the internet.

The data collected from sources produced by indigenous organizations are compared with those that have been disseminated by the Federal Government. Especially with regard to the number of indigenous deaths caused by the pandemic, it appears that there is an underreporting by the Ministry of Health, which does not account for the indigenous people living in the city. In addition to a quantitative analysis, these data reveal important questions about how the current government has addressed the issue of identity and the specific public health policies that should be aimed at these populations. The sources referring to the lives, letters, lawsuits filed with the Supreme Federal Court, websites and informational materials that have been circulating on the internet are analyzed from the indigenous reading and the decolonial criticism carried out to the current situation of absence of intercultural dialogue.

The methodological position adopted by APIB brings within the action and decolonial perspective by reaffirming the principle of self-identification of indigenous peoples, in a clear clash with essentialist and ethnocentric perspectives constantly formulated and reaffirmed in the context of current public policies. The denial of the identity of indigenous peoples translates as a reproduction of a monotopic vision of politics, society and law inherited from our colonial past, as the speech of the then Minister of Education, Abraham Weintraub, expresses so clearly:

I hate the term 'indigenous peoples', I hate that term. I hate it. The 'gypsy people'. There is only one people in this country. They want it, they take it. They don't want it, they may go backwards. They are Brazilian people, there is just one. It can be black, it can be white, it can be Japanese, it can be descendant of an Indigenous, but it has to be Brazilian, man! Put an end to this business of peoples and privileges (Leitão 2020).

In this sense, the theoretical and methodological references of the Modernity/Coloniality/Decoloniality movement are taken into consideration to be the most consistent with the picture described. The key concept of 'decoloniality', elaborated by Aníbal Quijano at the end of the 1980s, establishes an intrinsic relationship between the processes of global capitalist expansion, once started with European control over the Atlantic, and the racial classification of the various peoples that inhabit the world. This world-system, based on the subalternization of colonized territories and colonized inhabitants, structures a pattern of power that does not reduce the economic order, but violently affects all the epistemic universes affected. Thus, the independence processes of the former colonies did not annihilate the western referential patterns in world organization and understanding (Quijano 2005).

This concept of decoloniality has been unfolded by several thinkers: Edgardo Lander (Lander, 2000) who systematizes the coloniality of knowledge and the coloniality of nature; Maldonado Torres (Maldonado-Torres, 2007) who talks about the coloniality of being; and Maria Lugones (Lugones, 2008) who deals with the coloniality of gender. These discussions are also dealt with in the areas of pedagogy and interculturality by thinkers like Catherine Walsh (Walsh, 2005; Walsh, 2009) and Vera Maria Candau (Candau, 2000).

We also assert that several indigenous thinkers and activists have dialogued with this reference of research production and social action, precisely because they understand that it can be a possible way of communication of knowledge between universities and the references of other cultures, besides Western ones. As Ailton Krenak said, it is necessary "to understand what sharing this knowledge represents, having as protagonists the voices that come from these silent places" (Serena 2020).

Even though in this article we are concerned with describing the Brazilian indigenous realities in the current context, where the pandemic is on the rise and ethnocidal political discourses are emerging, we will also describe the indigenous reality in other periods and epidemiological contexts, indicating that this problem accompanies these peoples throughout their historical colonial and post-colonial contact. From the point of view that coloniality has its reverse face in decoloniality, we will present the actions and networks of solidarity organized by the indigenous peoples of Brazil who come, as Ailton Krenak wisely described, "stubbornly resisting the unsustainable embrace of progress" (Krenak 2017, 30).

Accordingly, this article aims to expose and discuss: the epidemiological history in the indigenous communities through the fronts of expansion and colonization; the indigenous conception of health that does not separate biological aspects from social ones, so that body, food, spirituality and nature are interconnected; the federal actions to confront the pandemic and its incidence in indigenous communities; the various indigenous strategies, which are articulated to other institutions, destined to confront and notify deaths and cases through the omission of the federal government: APIB and its grassroots organizations; ISA; Desai's; national and international NGOs, 16th ATL, Xingu Project, Fiocruz among others and, finally, the social impacts of the pandemic from the indigenous perspective and articulation with decolonial criticism.

## Results

### Epidemiological Background in Indigenous Communities Through Expansion and Colonization Fronts

Recent researches have shown that indigenous peoples have a high vulnerability to acute respiratory infections. In addition to past evidence that measles, smallpox and influenza viruses have culminated in major epidemics and consequent extermination of native peoples in Brazil, today "the introduction of respiratory viruses into susceptible indigenous communities has a high potential for spread, resulting in high rates of attack and hospitalization, with the potential to cause death"(Fiocruz, 2020; Valverde, 2020) and, similarly, cause exterminations such as those that and, in the same way, provoke exterminations like those that occurred during centuries of colonization in Brazil. There have been countless occasions of intentional contamination of these peoples by agents of expansionist fronts at various times in history. Invasions and epidemics are events with which indigenous peoples must deal since the maritime expansion and subsequent invasion and territorial conquest of vast regions of the Americas in the 16th Century onwards, occupied densely by a myriad of peoples constituting a multiethnic and linguistic diversity since at least 12,000 years A.P. (Carneiro, 1992, 42).

Pathogenic agents such as smallpox, measles, pertussis, chickenpox, typhus, diphtheria, flu, bubonic plague, malaria and, currently, Covid-19, are vectors for biological cataclysms in which indigenous peoples have been and are most affected, not only by lack of immunity, but also by other ecological, social and political factors. This fact exposes the illegality and criminality of the pandemics, bearing in mind that "the microorganisms did not affect a social and political vacuum, but a socially ordered world" (Carneiro, 1992, 13). This situation leads to impacts, not only biological, but also cultural and social, imposed by the issue of the rupture concerning the cultural reproduction of native peoples, given the greater vulnerability - in the case of the Covid-19 pandemic - of the elderly groups, who in these cultures represent the guardians of memory. Knowledge, wisdom, cultural heritage and ancestry are at risk (APIB 2020c) "Emergencia Indígena" 2020, 17).

The social and cosmological disrupting imposed on indigenous people since the beginning of coloniality, in policies of population concentration in Jesuit mission villages, and perpetuated in the action of the colonizers on expansion fronts, was responsible for the casualties of indigenous contingents in various parts of Portuguese America. The colonizing action begun in the coastal areas of Brazilian territory, extended to the interior regions, a place of ancestral experience of numerous indigenous peoples, most of them of Jê origin as opposed to the Tupi peoples of the coast.

At the threshold of the 16th Century, in spite of the controversies concerning the demographics of the native peoples before the invasion of Europeans in Brazilian territory, there were about 1–6.8 million indigenous people in the Amazon, Central Brazil and the Northeast coast (Carneiro, 1992, 14). In addition to this population density, which, according to the chroniclers of the time, was in a process of rapid expansion, it was visibly healthy and long-lived (Hemming, 2007, 216–217). Over the centuries, this dense population suffered processes of genocide caused by Europeans (carriers of microorganisms), making epidemics one of the main extermination agents of native peoples since then, portraying the stories of native peoples as stories of depopulation, deception and misunderstanding (Vainfas, 2007, 39). From a demographic index varying from 1 to 6.8 million in the year 1,500, this index gradually declined in the following centuries with figures of approximately 4 million in 1,600; 2.5 million in 1,500; 1.5 million in 1,800 and less than 1 million in the 2010 census (Santos, 2013, 185), due to the rapid spread of epidemics among the original peoples, whether today or in previous centuries.

Among the causes of death of the indigenous people contacted and resettled by the Jesuits at the beginning of colonization is the impact of epidemics caused by diseases from Eurasia and Africa, especially in Bahia (1,552–1,561) on the island of Itaparica (1). 562) and Ilhéus (1,563), besides the neighborhoods of São Paulo (1,554), Espírito Santo (1,559) and Pernambuco, in a process that triggered overwhelming levels of mortality "up and down the coast, penetrating deep into the interior" (Hemming, 2007, 223), in the 16th century. The same century that, according to hypotheses, the Goyá (an indigenous people whose ethnonym was an inspiration for naming the state of Goiás, in the Midwest region of Brazil) would have been extinguished by "successive outbreaks of cholera" (Quintela, 2003, 168).

Invasions resulting from organized military expeditions to capture indigenous people and search for gold, often following the indigenous trails towards the interior of the territory, have provoked conflicting inter-ethnic contacts with irreparable damage to the original peoples.

There were population decreases that would be fed back by the decreases promoted by the colonizers in search of more waves of indigenous people to fill the vacancies left by the previous groups exterminated due to the diseases that raged in the villages, in a repetitive process of gathering, contamination and deaths. In addition to the evangelization and integrationist intention of the Jesuit activity, simultaneously resumed in the politics of the current Brazilian Government, the villages fulfilled the function of liberating the spaces inhabited by the original peoples to the fronts of agricultural and extractive expansion. Besides, this situation imposed them a constant process of social and cosmological adaptability through the results of intrusions into their territories, inter-ethnic conflicts, illness and exterminations due to the viruses carried by the colonizers.

For a specific example, historiography shows that between 1751–1753, the Akroá, villages in São José do Duro, in the north of the Capitancy of Goiás, in Central-western Brazil, had their population drastically reduced due to the measles epidemic, with 500 people dying from 600 villages (Silva, 2006, 130). Similarly, data organized by Doctor Douglas A. Rodrigues (2013, 14) inform that only at the end of the 19th and 20th centuries, many indigenous peoples had high demographic losses caused by various epidemics when in contact with non-indigenous people, circumstances that allude to the genocidal process experienced by indigenous peoples in these 520 years of coloniality.

The Munduruku, in 81 years (1875–1956), had a 93% decrease in its population; the Kaigang of São Paulo, in 44 years (1912–1956) lost 92.7%; the Nambikwara in 8 years (1948–1956) lost 90%; the Asurini in 9 years (1953–1962) lost 81.5%; the Xokleng of Santa Catarina in 2 years (1941–1943) lost between 73.5 and 82.3% and the Karajá in 16 years (1940–1956) lost 75%, just to depict some of the cases. All these high mortality rates were driven by diseases such as smallpox, malaria, flu, measles, blenorrhagia, tuberculosis, chickenpox, whooping cough, and gonorrhea, whose degree of spread among the indigenous people is similar to the high rate of contamination by Covid-19.

Still in the 20th century, more precisely during the period of the civil-military dictatorship (1964–1985) in which the *modus operandi* of the Brazilian State was the liberation of indigenous lands - considered an obstacle to national development - to the execution of large undertakings including the construction of infrastructure works (highways, among them the Transamazonica and Hydroelectric Power Plants), there were deliberate contamination practicies, which means, criminal, of indigenous people by infectious diseases occurred (Ministério Público Federal "Figueiredo Report" 2020, 223). In this period, the Panará lost 82.4% of their population in 8 years (1967–1975); the Waimiri-Atroari 76.8% in 15 years (1971–1968); the Awá-Guajá from the Alto Turiaçu 72.5% in 5 years (1976–1981); the Parakanã 52.3% in 2 years (1970–1972), for diseases such as influenza, malaria and others^1^.

The spread of these diseases occurred not infrequently due to negligence on the part of the state indigenist agency at the time, as in the case of the Tapayuna people in western Mato Grosso "who, in 1969, allowed the participation of a journalist with the flu in the expedition led by the indigenous expert João Américo Peret, without the necessary prior vaccination for contact situations" Report of the National Truth Commission (2020, 25). The result of this neglect was a reduction from 1,220 people in 1960, according to FUNAI, to 40 people twenty years later.

In the 1980s, state negligence became frighteningly evident from the issue of mining on indigenous lands like the Yanomami, which had their territory invaded since 1975 and worsened in 1986. According to the Report of the National Truth Commission (2020, 30), the presence of approximately 40,000 miners on Yanomami indigenous land triggered a devastating impact of thousands of indigenous deaths (about 1,800 people between 1986 and 1990, representing 20% of the population (Oliveira, 2020)) and the resulting disappearance of entire communities. The situation portrayed by Davi Kopenawa and Bruce Albert in The Fall of Heaven: Words of a Yanomami Shaman.

Once a pandemic on March 11, 2020 was declared by WHO (World Health Organization), contamination by Covid-19 among indigenous peoples began precisely among the peoples of the Amazon, with the first confirmed record of contamination among the Kokama, on March 25, 2020, in the municipality of Santo Antônio do Içá. And the first record of death occurred among the Borari, on March 20, 2020 (APIB 2020c, 7–8), although the official record states that the first infection by the new coronavirus among Brazilian indigenous peoples occurred on April 2, 2020. Regardless, these data reinforce that the state of the Amazon is the region with the highest incidence of deaths among indigenous people. As of June 22nd, for example, the data indicated more than 5,000 confirmed cases of contamination and 293 deaths (APIB 2020c, 7).

The current government excludes indigenous people from urban areas or isolated territories from this accounting, which, according to APIB, represents institutional racism. It was necessary to monitor cases and deaths by the indigenous themselves through their associations in partnership with indigenous and health agencies partners with the creation of the Indigenous Emergency portal (APIB 2020c). In the North region, the contagion was caused by members of the SESAI (Special Secretariat of Indigenous Health) and the Brazilian Army, contaminating the regions of Alto Solimões (AM), Tumucumaqui Park (PA/AP), Vale do Javari (AM) and Alto Purus (AC).

On June 17, 2020, El País brought in a report by Joana Oliveira, records of contagion among the indigenous peoples of the Amazon interspersed with speeches by their leaders Kanamari and Marubo, whose pronouncements refer to the vulnerabilities potentiated by health agents and the military when inserting the new coronavirus into the villages (Oliveira, 2020).

Furthermore, specifically in the Javari Valley, extreme west of the Amazon, where the greatest concentration of isolated peoples and recent contact occurs, the registration of contagion occurred on June 4, by four employees of the DSEI (Special Indigenous Sanitary District) Javari Valley, with the rapid and gradual spread of Covid-19 among the Kanamari. On June 5, there were 3 records and between June 9 and 10, the numbers indicated 16 confirmed cases in two neighboring villages, in a trigger of fear between them, according to the words of Higson Kanamari, local leadership, recorded by Joana Oliveira of El País newspaper on June 17, 2020 (Oliveira, 2020).

It's scary. Many families took their children and fled the village, went to the head of the stream and we don't know anything else about them, we don't know if they are well, if they have been cared for. There is a village further up that made a barrier to not enter anyone from outside and to not leave. The thing is spreading very fast, and we have no hospital support near the village [more than a thousand miles away from Manaus. I fear for the isolated peoples of the region, who are even more vulnerable.

In the Indigenous Land of Tumucumaque Park, the border region of Amapá and Pará with Suriname, the indigenous leaders denounce that the military took the new coronavirus to the region, where there are at least 23 infected, including a five-month pregnant woman transferred in a serious state to Macapá. The first two were indigenous people from the Tiriyó Mission village who work in a outsourced company serving the first Special Border Platoon (first PEF), where about 50 military personnel from the Army and the Brazilian Air Force (FAB) work.

This is a region with six indigenous peoples and at least two isolated, totaling 1,700 people. The denunciation was contested by the Ministry of Defense. In addition to health professionals and military agents, the virus has reached these regions through invasions of indigenous lands undertaken by miners, grill workers, missionaries, fishermen and illegal hunters during the pandemic and also through contaminated indigenous people seeking emergency assistance in the cities (Socio-environmental Institute 2020; Socio-environmental Institute, 2020a).

After months of living in the forests to flee from coronavirus contagion, many indigenous people faced the need to obtain supplies for which financial support was essential, as Dario Vitório Kopenawa, vice president of the Hutukara Yanomami Association and Adelson Nascimento Bolivar, indigenous leader of the Mafi community on the Cauaburis River, respectively, reports:

This benefit matter does not let people stay in the villages. They need some objects like tobacco, shorts and nets. Sometimes it gets in the way of this crisis scenario. It is a very difficult problem. It hinders social isolation. They want to buy something, needing something in the village: soap, soap, cutter. We need to get food, salt and tobacco. It is more necessary for us to use. After 3 or 4 months in the area, we come to the city. I had only come here in January. I isolated there, but we ran out of salt. Then I can't stand it (Hamdan, 2020).

The exposure to the virus by indigenous people is a further neglect on the part of the Federal Government to establish a differentiated policy for indigenous peoples in the context of health (SESAI) and the economy (emergency financial aid to be drawn from bank branches in cities with high levels of contamination) (Hamdan, 2020). All these circumstances make the Amazon the area with the highest rate of registered cases, a worrying situation because it contains indigenous lands of difficult logistic access and with a higher incidence of peoples in voluntary isolation and recent contact (APIB 2020c, 14).

The contamination on indigenous lands in Mato Grosso do Sul (Center-West region), western Paraná, Santa Catarina and Rio Grande do Sul (Southern region), on the other hand, was caused by indigenous employees of the region's agribusiness represented by companies in the frigorific sector (APIB 2020c, 18).

Since then, hundreds of people have been suffering from the spread of Covid-19, in a process of interiorization of the new coronavirus, in the five Brazilian regions, imposing challenges to indigenous and non-indigenous researchers from various areas and institutions and to the indigenous movement itself, especially the APIB. Of the 305 indigenous people in Brazil, according to APIB data as of September 1, 2020, 156 have already been affected by the Covid-19 pandemic (APIB 2020d). In addition to the vectors for the spread of the new coronavirus (invaders of indigenous lands), the way of life in the villages, with extensive family models, object sharing and continuous contacts contribute to the rapid spread of Covid-19 (Rodrigues, 2013). Several actions and strategies were and continue to be discussed, as a matter of urgency, and put into circulation in the national and international context through social networks given the context of social isolation required by the pandemic.

### Indigenous Health: Body, Territory and Spirituality Interconnected

In addition to the questions above mentioned, within the realities of many indigenous peoples, their medicinal knowledge conflicts with the knowledge of biomedicine. Many health professionals do not understand the traditional indigenous actions and treatments, which in large part come from cosmological visions.

The indigenous conception of health does not separate biological aspects from social aspects, so that body, spirituality and nature are interconnected. In order to go a little further into this issue, we will cite two indigenous realities that occurred in the 1990s and 2000s: the Siona and the Upper Xingu indigenous peoples. Later, we will mention the conceptions of the Huni Kuin about the Covid-19 today.

### Siona Indigenous People

The Siona are one of the Tucano indigenous people, inhabitants of the Amazon Forest in Colombia. Within the search for health and cure, the interpretations and meanings attributed to diseases are constantly negotiated. The flu, pertussis and measles decimated approximately 75% of the Siona population between 1900 and 1925, soon after the construction of roads aimed at the installation of an oil industry and the emergence of the small town of Puerto Asís, 45 km from the Siona reserve.

The presence of non-indigenous people made several healing alternatives introduced by the official biomedical service emerge. Despite the Siona's openness to other forms of treatment than their own, there have been no changes in the way they think about diseases or the processes of seeking a cure. Biomedicine alone has not proven effective (Langdon, 1994). The representation of diseases among the Siona comes from powers operating in the invisible world.

The Siona Universe is composed of five hierarchical levels, each of them populated by various classes of entities, such as the celestial figures (Sun, Moon, and thunder), human beings, animals, and evil figures (watí). For them, the world has two sides: "this side" of the visible world and "the other side" of the invisible world. Entities on both sides constantly influence day-to-day activities. It is the shamans who are responsible for maintaining the balance between the forces, since they are the mediators between "this side" and the "other side". To maintain this control and to "see" what is wrong with the "other side" that affects "this side", they drink a hallucinogen named iko, or yagé, or ayahuasca (Langdon, 1994).

Diseases can be caused by watí or by attacks from enemy shamans. Even if the disease was not caused by the invisible world it becomes necessary to find out what caused the disease in this world. If the cause is discovered, it occurs the removal of the disease or the object (materialization of the disease) of the victim in order to cure it completely. Siona's world and health vision can be apprehended by deepening four key concepts: wahi, hun'i, iko and dau. Wahí is opposed to *Huns*, the first meaning to be alive, healthy, strong, young, young and the second meaning the opposite (Langdon, 1994, 120). The *iko* refers to medicines, which can be hallucinogenic or not.

Most of the non-hallucinogenic remedies are used for the treatment of disease symptoms and can be ingested, applied to the skin or used in baths. Through the existence of the name Siona iko, there was no resistance to the incorporation of the treatments offered by biomedicine.

Many drugs are classified by them as iko and are widely used when needed and available. The term dau has three different uses: 1) as a substance that grows inside the shaman and endows him with powers and knowledge; 2) as a concrete substance that causes misfortunes; 3) as a disease (Langdon, 1994).

As previously mentioned, the Siona suffered a process of decimation due to diseases, mainly at the beginning of the 20th century. Nevertheless, instead of attributing the causes to "white disease," they attribute the causes to conflicts between shamans of rival groups who sent their *dau* to spread diseases from one community to another. The author describes the therapeutic itinerary of Julia, a Siona in search of cure. Julia has symptoms of swelling through her body and stomach. There are at least two causes for the disease: the *watí* (she went to the river in order to wash her clotes, also she was menstruated and was pushed by a *watí*) and the *dau* (sent by a shaman from another village in case of disagreement with her village). Her husband refused to take her to the doctors and so he continued taking care of Julia through shamanistic treatments, associated with the use of drugs (bought in the city's pharmacy) and mestizo treatments. Julia is taken to medicinal baths in hydrothermal springs in Mexico, but on her way back home she feels sick and ends up dying. As for the family, it was the shaman of the other village who killed her. They begin to accuse him of murder.

When the delegate inquires Julia's husband about the causes of her death, he answers that it was dropsy. In order not to be arrested, he attributed Julia's death to "white disease", making himself understandable within the Western system, even though he believed she died for a *dau.* Langdon, (1994) shows us that the Siona fluidly negotiate the causes of a visible and invisible world within their beliefs. No matter how much chemicals are used, hospital treatments combined with shaman treatments, the cure of the disease and the disease itself will always be attributed to the activity of the shaman.

### Indigenous People of the Alto Xingu

According to Novo, (2011), indigenous health in Brazil gained strength in public policy in the mid-1980s. At this time it also gained space for the health reform movement that had as a model the primary health care policy. The indigenous organizations emerged and developed, which further stimulated the debates, culminating in advances in the discussions regarding indigenous health. The author notes that at the First National Conference on the Protection of Indian Health, held in 1986, the foundations were established for the creation of a subsystem for indigenous health care, within the national health system, which proposed a model of differentiated care for the people.

In this context, the importance of indigenist movements that start using the space opened by public policies to claim autonomy and self-management of activities and resources is growing, and they propose that indigenous health is more than just a matter of sanitarianism and epidemics. Since the 1990s, a new form of public health policy was put into effect through the creation of the Unified Health System, of which indigenous health became part of (Novo, 2011).

Despite the advances, it was only in 1999 that an Indigenous Health Care Subsystem was established with the creation of 34 Special Indigenous Health Districts (DSEI) that should act as mediators between indigenous peoples and government bodies. In Alto Xingu region, with the establishment of these DSEIs, some conflicts related to the health management model and the knowledge of indigenous peoples have intensified. The author states that the Leonardo Indigenous Center, located in the Alto Xingu, created in 1946, depicts a privileged place of knowledge contact. It is the place where the information brought from the city is concentrated, meetings take place and where the multidisciplinary health team remains. It is also there that two distinct models of thinking about health are placed: the first is the sanitary model with the performance of non-indigenous professionals, and the second corresponds to the model of traditional therapies, linked to cosmologies and Xinguan political organizations. It is, therefore, in this space that the resignifications and oppositions of biomedicine and traditional indigenous medicine take place, in an attempt to build a hybrid system of treatments (Novo, 2011).

This possible hybridization and at the same time counterposition has occurred in the most diverse indigenous peoples, often provoking conflicts in relation to different expectations about public health policies, mainly because they are guided by technical notions based on biomedicine.

Novo (2011) states that when the Leonardo Center was built, it was considered by the indigenous people an inappropriate place for health. The problems reported by the indigenous people were several: it corresponded to a very cold place to spend the nights, because it was not possible to light fires due to the number of people staying and because there were patients with respiratory problems. Another issue concerns the hospitalization of patients, which implies forced coexistence between people of different ethnic groups and villages. According to Xinguan cosmologies, illness is an external aggression introduced into a person's body that can materialize into substance or objects, usually by the action of a spirit, witchcraft, or the evil action of other people (envy, jealousy, anger).

If there is concentration of people in a single place of hospitalization, there is the sharing of substances and the actions of each of the patients interfere in the process of illness or healing of others. Thus, the Leonardo Center imposes a coexistence between people that is dangerous because it prevents the fulfillment of restrictions (food, for example) and forces the approach of potential sources of illness (people from other ethnic groups and villages). In addition, the Leonardo Center is a place of concentration for health professionals, who for the most part are not predisposed to go to the villages to get to know the local realities, and this issue is the target of criticism by indigenous leaders. Often, the establishment of moral judgments regarding the behaviors and procedures of traditional indigenous medicine interferes with the relationships between indigenous and non-indigenous people, and the choices made for healing procedures.

Novo (2011) realizes that indigenous people seek Western health treatments as an alternative to traditional medicine, without leaving it aside. They remain with their own ways of thinking about the process of illness, healing and health treatment.

From these two realities, it is possible to perceive that national public policies conflict with the actions, ways of thinking and being of indigenous peoples. In the case of the Siona, the way of interpreting the illness contradicts the way the civil authorities (police and delegate) understand illness and death. In the case of the Xinguan peoples, the conflicts are related to the physical structure of the Leonardo Health Center and the political structure of health care. We understand that, even in the midst of conflicts, the forms of negotiation could be more fluid.

Nowadays in the case of health treatments for the indigenous people against the new coronavirus (Covid-19), there are constant clashes and negotiations between traditional indigenous and biomedical knowledge. According to Lagrou (2020), the perception Huni Kuin about the diseases and contagion by the new coronavirus are related to the forms of coexistence between humans and non-human. The Huni Kuin of the eastern Peruvian Amazon forest share the thought with other indigenous peoples of the region that diseases are caused by food, especially when we eat animals. People can get sick because the other beings we assault or interact with take revenge and send their *nisun*, pain and other symptoms that can result in illness and death. Only shamans, with the use of psychotropic plants, can discover the action of these invisible agents and act, through singing, the blowing of smoke from medicinal plants, to recapture the spirit of the victim. Hence, the rules of food and negotiation around hunting point to a knowledge about the pathogenic potential of animals. Respect is needed so that the hunt does not turn against the hunter. In addition, the Huni Kuin know that epidemics are also the result of the extractive relationship between cities and forests. It is not the fact that humans eat game meat that causes the epidemics, but the deforestation and extinction of animals that used to be their symbiotic hosts (Lagrou, 2020).

Below we will describe the various indigenous strategies for confronting Covid-19 in the current context, in view of the historically constructed problem of constant socio-cultural negotiations for the search of treatments and cure.

### Indigenous Strategies to Face the Pandemic

The first Indigenous Women's March, held in August 2019 in the federal capital city (Brasília), had as its theme "Territory, our body, our spirit". More than 2,500 indigenous women, many of whom traveled more than 2000 km, claimed the right to health, education, and the protection and demarcation of their territories. One of the movement's guidelines was to protest against the Ministry of Health's proposal to decentralize the management of the Special Indigenous Health Secretariat (SESAI), that is, to remove the Federal Government's obligation to offer these services, which would lead to immense losses of acquired rights (Roman, 2019). The final document of this march makes explicit the fact that the indigenous struggle for body health is linked to the health of the spirit and all forms of life that inhabit their territories:

We are totally against the narratives, the purposes, and the acts of the current government, which has been making explicit its intention to exterminate the indigenous peoples, aiming at the invasion and genocidal exploitation of our territories by the capital. This way of governing is like uproot a tree from the earth, leaving its roots exposed until everything is dry. We are stuck in the earth, for it is there that we seek our ancestors and for it that we feed our lives. Therefore, the territory for us is not an asset that can be sold, exchanged, exploited (APIB, 2019). The territory is our own life, our body, our spirit. (APIB, 2019)

The scrapping of indigenous health, which led the women at the march to occupy the SESAI building in Brasilia, indicated a situation that would worsen violently after the spread of Coronavirus in indigenous communities. Through the already reported neglect of the Brazilian Federal Government, the indigenous peoples of Brazil have created different autonomous strategies to combat the pandemic: creation of SOS in social networks; publication of information booklets in their mother tongues; production of lives; articulation with Universities, Museums and Research Institutes of the most diverse fields of knowledge; search for the support of international organizations such as the International Labor Organization (ILO) and the Inter-American Commission of Human Rights; production of videos; elaboration of journalistic denouncement; elaboration and sending of projects of fight against the pandemic to the Legislative Assembly; escape to the forest; isolation of the villages; raising of food and personal protection equipment, etc.

These actions, which have occurred according to the local and social reality of each indigenous people, converge their struggles for the Articulation of Indigenous Peoples of Brazil (APIB). The APIB created the National Committee for the Life and Memory of Indigenous Peoples in a meeting that took place between May 8 and 9, 2020. The committee integrates several regional indigenous organizations of the country as: Articulation of Indigenous Peoples of the Northeast, Minas Gerais and Espírito Santo (APOINME); Council of the Terena People; Articulation of Indigenous Peoples of the Southeast (ARPINSUDESTE); Articulation of Indigenous Peoples of the South (ARPINSUL); Great Assembly of the Guarani People (ATY GUASU); Coordination of Indigenous Organizations of the Brazilian Amazon (COIAB) and Yvyrupa Guarani Commission (Methodology of the APIB, 2020 network). In addition, the National Committee for the Life and Memory of Indigenous Peoples has the participation of indigenous health specialists from different civil entities.

The aim of the Committee is the formation of a national network of solidarity, collection of donations and formalization of denunciations of omission by the Federal Government. The actions are based on three main axes: to guarantee differential care to the indigenous peoples; to elaborate legal actions of political incidence and actions of communication and information in health.

One of the most important actions of the Committee has been the mapping of the dissemination of the coronavirus among the communities and the identification of the main vectors. As mentioned above, the researches carried out pointed out the Federal Government as one of the transmission agents of Covid-19, since the first case of indigenous death occurred due to their contact with health professionals. Another aggravating factor is the displacement of indigenous people to cities in search of emergency assistance (APIB, 2020d).

This national network that articulates the different contexts of Brazil's indigenous societies extends to include the indigenous populations of Latin America and other regions of the world. The letter addressed to the President of the World Health Organization, prepared by the Joint Parliamentary Front in Defense of Indigenous Peoples, calls for special attention "...to indigenous peoples around the world, guiding all governments specifically on ways to guarantee the rights to life and health of indigenous peoples and articulating with other competent entities the policies to guarantee". (Socio-environmental Institute, 2020a):

1. The physical, territorial, food and cultural security of indigenous peoples;
2. Recognize the greater vulnerability of indigenous populations to the pandemic;
3. Ensure access to inputs for personal hygiene, basic sanitation, social rights and social security, diagnostic testing, respirators and personal protection equipment;
4. Territorial protection and guarantee the right to social isolation through the prohibition of loggers, miners and other fronts of expansion on their lands;
5. Guarantee the participation of indigenous organizations in the planning of actions to combat Covid-19.

These same demands are found in Bill no. 1,142 of 2020 (Federal Senate, 2020) aimed at creating an Emergency Plan to Confront Covid-19 in Indigenous Territories, Quilombolas and other traditional communities. The project was approved by the Federal Chamber on May 21 and by the Brazilian Senate on June 16. Only on July 7, President Jair Bolsonaro sanctions the new law, however, vetoes 22 items of it (APIB 2020b). Among the vetoes we highlight: Specific emergency budget allocation; creation of a specific credit program for indigenous peoples; two devices that gave a period of ten days for the development of a contingency plan for each situation of contact with isolated peoples; universal access to drinking water; free distribution of hygiene, cleaning and surface disinfection materials; emergency supply of hospital beds and Intensive Care Unit (ICU); acquisition of ventilators and blood oxygenation machines; distribution of information materials about Covid-19; and internet points in the villages (Senate Agency, 2020).

On July 8, the Brazilian National Congress overthrew 16 presidential vetoes. However, some were maintained that, if overturned, would be essential for the health and safety of indigenous peoples, such as: release of emergency funds for indigenous health; distribution of basic food baskets, seeds and agricultural tools; creation of a specific credit program for indigenous and traditional populations. Representative of the project in the Legislative Assembly, Joenia Wapichana^2^ said that " the overthrow of these vetoes means protection of the lives of indigenous peoples. From this moment on, we have a collection tool, including a judicial one, in case there is no respect for the implementation" (Cofen, 2020).

Taking up the demands of the Indigenous Women's March, indigenous health requires coordinated actions. For example, the indigenous people demand the right to drinking water, which will occur when there is an immediate withdrawal of the miners who exponentially invade their territories and promote an aggressive intoxication of the rivers, groundwater, and all life in the region. It is necessary to admit and take energetic measures against the fires that have weakened the respiratory system of the forest people. Even if some ministers support disintrusion, the main argument is that it is not appropriate to carry it out at this time, as Minister Luis Roberto Barroso says "no one should imagine that 20,000 people with a snap of their fingers or a pen are withdrawing from just one of the seven indigenous communities. Planning is necessary, not least because no one wants an armed war within the indigenous community; a plan is needed" (Souza and Vasconcelos, 2020). So far, the approval of Bill no. 1,142 of 2020 and the precautionary measure combine the diverse efforts of indigenous entities and all supporters of the movement to combat the pandemic among the most vulnerable peoples.

### Analysis of the Social Impacts of the Pandemic from Indigenous Perspectives and Articulation with Decolonial Critique

The impacts of Covid-19 on indigenous populations are numerous. There are impacts in relation to the lack of clarity regarding the protocols of the Ministry of Health, which is being denounced by indigenous leaders. The protocols regarding prevention, care, and burial actions clash with indigenous traditions. The absence of intercultural dialogue and the epistemic and ontological violence being reproduced by the Brazilian State is clearly perceived. The cases of the Yanomami and the Wai Wai can be highlighted. On June 24, 2020, El Pais published a report on Yanomami mothers who claimed the bodies of their babies contaminated by Covid-19:

Three women face such terror for which it will be necessary to create a name. They are Sanöma, a Yanomami group, and their village, Auaris, is in what the whites call Roraima, on the border between Brazil and Venezuela. They do not understand the idea of a border, for them the land is one - and has no fences. They don't speak Portuguese, they speak their own language. In May, these women and their babies were taken to Boa Vista, the capital of Roraima, suspected of pneumonia. In hospitals, the children would have been contaminated by Covid-19. And they died there. And then their little bodies disappeared, possibly buried in the city cemetery. Two of the mothers are with Covid-19, crowded in the Casa de Saúde Indígena (CASAI), full of sick people. There, devastated by the virus, they beg for their babies (Brum, 2020).

The report highlights that in addition to being torn from their villages on suspicion of pneumonia, these mothers and their babies suffered contagion in the hospital. Eliane Brum (2020) points out that for the Yanomami a body should not be buried but cremated after a long ritual. At the virtual meeting of the Joint Parliamentary Front in Defense of the Rights of Indigenous Peoples on July 13 this year the Wai Wai leaders claimed the bodies of their relatives who died as a result of Covid-19 and who were not returned to the village for burial for the Wai Wai rituals (Virtual Meeting, 2020).

They were buried in Boa Vista, the municipality where they were hospitalized. This situation was questioned to the coordinator of DSEI-leste and a complaint was forwarded to the Public Prosecutor. Some indigenous leaders mentioned that many Wai Wai do not want to be taken to the municipalities fearing they will die and not have their bodies removed to their villages for proper cultural treatment, another complaint is about mistreatment. Therefore, there is a lack of respect on the part of the DSEIs for the constitutional provision of differentiated health for indigenous people in view of the cultural singularities and historical vulnerability that impacts the issue of ethnic survival itself.

The total absence of inter-culturalism in public agencies directed at indigenous issues already begins with the appointment of managers that goes back to an idea of indigenous mentoring. At the aforementioned meeting of the Mixed Parliamentary Front, the Attorney General in Boa Vista, Alisson Murungal, pointed to the militarization of the management in SESAI and the DSEIs. There is no presence of indigenous people in these organs. Mário Nicácio of COIAB, commented that he is indigenous, lives in the Amazon and is not part of the Amazon Council which also has as president an ex-military, the vice-president Hamilton Mourão. The mentality of the times of the Military Dictatorship in Brazil present in these organs is very far from being compatible with intercultural debate. Referring to these managers, Dario Kopenawa Yanomami informed that they have never stepped on indigenous territory to know the conditions of the indigenous. He also mentioned the precarious health structure, that the professionals there are working under tarps and emphasized that because it is a very mountainous territory access is extremely difficult. Among the indigenous people there are health impacts due to the lack of medical assistance^3^ and basic hygiene materials, access to drinking water, hospital beds, medicines and food security.

Along with this serious situation, President Jair Bolsonaro defends the preventive use of unproven drugs for Covid-19 among indigenous populations. This situation was denounced by the indigenous peoples, asking for preventive measures against a possible genocide promoted by the Federal Government, since they would be used as guinea pigs for drug testing, and also used for political purposes to favor agribusiness and mining, since there would be illegal takeover and invasion of their lands during this moment of extreme fragility.

We have observed that there is a clear disposition of the Federal Government in the economic use of indigenous lands, which reflects in the paralysis of the demarcation processes and in incentives to invaders of indigenous lands, including with the use of legal measures such as the Provisional Measure 759.

It is important to mention that one of the deep impacts of the Covid-19 pandemic on indigenous peoples concerns their ancestral memories. Since this is a disease that affects mainly the most vulnerable such as the elderly, it is especially impacting among indigenous people, since in the dimension of orality the elderly are the guardians of memory and culture.

Another issue that should be addressed to when thinking about the impact of a pandemic on indigenous populations is history. Historically, the indigenous have gone through several situations of epidemics that since the contact with the colonizer were responsible for genocides of a large part of this population. Now, from APIB data it can be observed that indigenous populations in Brazil are more vulnerable to the pandemic than the average non-indigenous population.

Data from the Ministry of Health point out that, among the Xavante, the lethality rate of Covid-19 is 160% higher than the average rate of the Brazilian population. Bruno Ribeiro's article in the PDT page reveals that if the average of the general population is 4.5%, the indigenous population is higher reaching 6.8% and that in the Xavante Namurunja village in Barra do Garças in Mato Grosso this number has a worrying record, since it is 11.7%.

If compared to the number of dead Indians in the whole country, the index with Xavantes shows an even more worrying scenario. According to the Amazonian Environmental Research Institute (Ipam) and the Coordination of Indigenous Organizations of the Brazilian Amazon (Coiab), the lethality rate with Indians in Brazil is 6.8%. The figures therefore show a variation of 72% (Ribeiro, 2020)

Rafael Were, who is Xavante and national president of the Indigenous Movement of the PDT, points out that the vulnerability of indigenous populations is intensified by the genocidal policy of the Federal Government. He mentioned the lack of medical assistance, ICU beds, difficulty in locomotion, invasions and deforestation as situations responsible for the aggravation of contagion and deaths among the Xavante. In June 2020, the Namurunja village of approximately 22,000 indigenous people witnessed the deaths of their relatives, among adult children and the elderly, including leaders.

In the midst of the pandemic, most villages reinforce traditional medicinal treatments, associating with them conventional biomedical treatments. In an interview to the Nexo newspaper, the coordinator and treasurer of COIAB, Angela Kaxuyana, of the Kahyana people, revealed that:

In the face of diseases and before performing laboratory tests, [the Munduruku] always prefer to consult with them, because the diagnosis of shamans is usually more relevant. Besides the knowledge of traditional remedies, their recommendations go beyond the physical-material level, making reference also to diseases projected by the 'strength of the supernatural', connecting the present with their myths (Rocha, 2020).

At the virtual meeting of the Joint Parliamentary Front on July 13, Aldenir Wapichana together with other indigenous leaders mentioned that much of Covid-19's chartering has taken advantage of the traditional medicinal knowledge of their cultures Virtual Meeting, 2020. Therefore, it can be observed that the pluriepistemic dialogue is fundamental for the confrontation of Covid-19 not only for the indigenous populations, but for the society in general.

## Discussion

This article described and analyzed the current situation of the Brazilian indigenous peoples' confrontation with Covid-19. We aimed to present a critical overview of the main problems that emerge in the daily scenario and the political and social struggles faced by the country's indigenous peoples. From the data presented, we clearly perceive the absence of intercultural dialogue, epistemic and ontological violence being produced by the Brazilian State in the midst of the crisis against the Covid-19 pandemic. Among the absences of dialogue and violence we can feature, may include: lack of health care; facilitation or neglect of intrusion by invaders of indigenous territories; lack of respect for indigenous customs, their ways of being and acting; among others.

Once we review the question raised at the beginning of this discussion, we realize that in order to seek a possible symmetrical dialogue between the indigenous people and the Brazilian public power, it is necessary to spend more than changes in attitudes; it is necessary to change the way one perceives the other. We reinforce the need to listen and understand the other, considering in fact their way of thinking and acting, according to their beliefs and relationships that they establish with the community (be it their family or their broader social group). And we emphasize the need for hybridization of practices so that they have real chances of working, with less subordination of knowledge and more dialogue.

Furthermore, we observe that, for the indigenous populations, the confrontations with the Covid-19 pandemic are amplified, either by the historical relationships of violence, or by the violations and crimes committed by the Brazilian State in its current configuration. Indigenous organizations and leaders are mobilized to demand protective measures from the State. There are countless confrontations with historical and institutional racism within a state policy that clearly aims at eliminating indigenous ways of life for the economic exploitation of their traditional territories.

One point of great importance in this confrontation concerns the production of information and communication, and in this context we have seen the initiatives of indigenous organizations such as the radio program Coiab informs. In the 14th edition, the international day of the Amazon was highlighted. This edition drew attention to the resurgence of violence by the current government and denounced its genocidal and ecocidal policies. According to the edition, after six months of pandemic, there were no effective measures to reduce the contagion of the disease among indigenous people and, on the contrary, what is seen by the government is the legalization of crimes within indigenous lands with the increase of invasions of territories.

Issue 7 dealt with the first meeting on the situation for the protection of indigenous peoples in voluntary isolation and those in recent contact. It brought the lines of indigenous leaders such as Sonia Guajajara and Angela Kaxuyana. These lines expose how the government has treated the Covid-19 agenda among indigenous peoples. Angela Kaxuyana revealed that she had her microphone blocked and that she and other indigenous leaders were intentionally prevented from speaking. The indigenous leadership regreted the fact that the State is against indigenous constitutional rights and is not open to discuss, let alone listen to, indigenous peoples in their own demands.

According to data from SIPAD (Inclusion Superintendence, Affirmative Policies and Diversity), indigenous leaders, associations and entities warn of the delay in sending assistance to villages and mobilize themselves to access resources to combat and prevent COVID-19. Many peoples have gathered to form a support and solidarity network and disseminate information in indigenous languages through booklets, videos (bilingual audiovisual) financial and food collection campaigns. In addition to these, lives on Facebook, Instagram, Youtube, radio programs, such as those broadcasted by Rádio Yandê, have been important tools in the dissemination of information to combat the pandemic. The campaigns have recommended social isolation with the sympathetic statement “Stay at home, kin”, explaining the ways of transmission and preventive procedures for non-contamination, such as avoiding as much as possible the circulation of people, if necessary to make community purchases, sanitize food before entering the villages, to consume food from the village itself, encouraging their own food production. They also recommend that they should not leave their villages, as in the city they are more vulnerable to contamination, and if they die there is an attempt to hide and make indigenous identity invisible.

As a result, such diseases have historically been weapons for the extinction of indigenous ways of life and the Covid-19 clearly reveals the asymmetry of intercultural dialogues. On the part of the State and of Brazilian society we have cultural deafness, on the part of indigenous populations we have interculturality as a way of resistance and to make society aware of the importance of intercultural and pluriepistemic dialogues as a form of human survival, as stated in the final letter of the Assembly of Indigenous Resistance: “In times of pandemic the struggle and collective solidarity that has rekindled in the world will only be complete with indigenous peoples, because the healing will not only be in the active principle, but in the activation of our human principles” (APIB, 2020e).

## Data Availability

The original contributions presented in the study are included in the article/Supplementary Material, further inquiries can be directed to the corresponding authors.

## Footnotes

1 Also check the video *Indigenous Health in Times of Pandemic and the initiatives of indigenous peoples of self protection*. https://www.youtube.com/watch?v=3JDf2mSGWaE

2 Joênia Wapichana was the first indigenous woman to take a seat in the Brazilian National Congress.

3 The health issue among the indigenous has been getting worse since the inauguration of the current president, Jair Bolsonaro, responsible for the end of the Brazilian National Mais Médicos Program.
